# Pennsylvania

**Published:** 1896-07

**Authors:** 


*Pennsylvania.* A herd at Economy, in this State, have shown some discrepancies in the second test, which has given rise to some differences of opinion among the examining veterinarians as to the relative value of the preparations of tuberculin used for diagnostic purposes. It seems to be another instance where a second test revealed a smaller number of cases as indicated by the reactions, which is quite in keeping with the more extended knowledge we are gaining of these agents used for diagnostic purposes, but which seem to have some additional value as curative or palliative influences for a variable period of time.

A herd of fourteen cattle condemned as tuberculous were recently destroyed near Doylestown, Bucks County, under directions of the State Live-stock Sanitary Board.

